# The Reductions in Phenomenology - A Comparison Across Main Authors

**DOI:** 10.1192/j.eurpsy.2024.1372

**Published:** 2024-08-27

**Authors:** N. D. Ramalho, I. Lopes, T. Rocha, G. Santos, J. Leal, J. F. Cunha, D. Seabra, D. Santos, J. C. Moura

**Affiliations:** ^1^Psychiatry and Mental Health Department, Centro Hospitalar Barreiro Montijo, Lisbon, Portugal

## Abstract

**Introduction:**

Phenomenology is one of the fundamental tools in the clinical practice of psychiatrists, constituting one of the touchstones regarding the diagnostic framework in which clinicians navigate.

For Husserl, Phenomenology provided access to the structure of pure consciousness, experience and existence. These are conditions of possibility for the object of Psychiatry, ontologically prior to it. Thus, clarification of the object and method of Phenomenology is preliminary to understanding the object of Psychiatry.

Phenomenology, being a direct tributary of Philosophy, evolves dialectically, constantly dialoguing with its predecessors. While it is taken as a philosophical current, it is also considered a method. It is precisely as a method that we can see how the methodology changes in different phenomenological traditions.

**Objectives:**

To compare how the main phenomenological traditions operate.

**Methods:**

Comparative analysis between the phenomenological reductions in key figures of the phenomenological tradition, resorting to the corpus of the *Husserliana*, *Being and Time, Phenomenology of Perception* and *General Psychopathology.* Additionally, a non-systematic literature review of papers on the database Philpapers, using the keywords “critical phenomenology”, “eidetic reduction”, “phenomenological reduction”.

**Results:**

While there is a multiplicity of ways of taxonomizing phenomenological currents, we divide it in: pure, existential, embodied, jasperian, psychopathological, and critical.

Husserl’s pure phenomenology uses the free variation in phantasy and *epoché* as operators, starting from the natural attitude.

Heidegger’s existential phenomenology makes no reference to a reduction of any kind. For him, it is necessary to take a step back, to a more primordial mode of being through which we can access Being, where the world is given and constituted.

Embodied phenomenology, of Merleaupontinian provenance, recognizes the reduction, but cannot be fully achieve it.

Jasperian phenomenology uses empathy and co-experience as its operators, through which it gains access to the subjective states of the other, with the aim of systematizing and taxonomizing subjective phenomena.

Phenomenological psychopathology tentatively uses Husserlian reductions to identify the a priori structures of the human, be it Biswanger’s forms of manifestation of failed human existence or Blakenburg’s anthropological disproportions.

Critical phenomenology uses a historical-transcendental analysis of experience as its operator, through which it accesses transcendental intersubjectivity.

**Conclusions:**

At a time when the DSM and ICD are increasingly seen as inadequate, limited and dogmatic, the resurgence of interest in Phenomenology is evident. It is important to avoid falling back on new presuppositions without constant revision and questioning, at the risk of simply mutating dogmas and missing the original legacy of pure phenomenology, the suspension of presuppositions.

**Disclosure of Interest:**

None Declared

